# Balloon dilation of the eustachian tube using endovascular balloon under local anesthesia—a case series and systematic literature review

**DOI:** 10.3389/fsurg.2024.1271248

**Published:** 2024-02-20

**Authors:** Omer J. Ungar, Münir Demir Bajin, Valerie Dahm, Vincent Y. W. Lin, Joseph M. Chen, Trung N. Le

**Affiliations:** Department of Otolaryngology, Head & Neck Surgery, Sunnybrook and Women's College Health Sciences Centre, University of Toronto, Toronto, ON, Canada

**Keywords:** endovascular catheter, endovascular balloon, balloon dilation, eustachian tube, eustachian tuboplasty, eustachian tube dysfunction

## Abstract

**Objective:**

To report a novel technique in Balloon Dilation of Eustachian Tube (BDET) using an endovascular balloon (EVB), in a prospective cohort. The results are compared with reported outcomes using standard balloons.

**Methods:**

Demographic information and clinical parameters were collected prospectively fora series of patients with obstructive eustachian tube dysfunction (OETD). Balloon dilation Eustachian tuboplasty was performed under local anesthesia in a tertiary referral center, using the EVB. Systematic literature review was used for comparison, using Medline via “PubMed”, “Embase”, and “Web of Science”.

**Results:**

Eight OETD candidates (12 ears) were enrolled; 5 males and 3 females. Average age was 48 (range −23 to 63) years. The most common presenting symptom was aural fullness (9/12), followed by ear pressure (7/12), hearing loss (5/12) and tinnitus (4/12). Otoscopically, tympanic membrane retraction was evident in 10/12 ears, the majority of which was class II—Sade classification. Pre-operative tympanogram was type B and C in 7 and 5 ears, respectively. All BDETs were performed without complications. Post-operative tympanometry was A in 8/12 ears. Post-operatively, Eustachian Tube Dysfunction Questionnaire-7 results reduced to within normal limits (average score ≤3) in 11/12 ears (*p* = 0.0014). The systematic literature review included 6 papers (193 patients, 262 ETs) with comparable results, most also with little adverse effects.

**Conclusion:**

BDET using an EVB is a safe and effective option for OETD. It is well tolerated under local anesthesia in properly selected individuals. The reduced procedural cost may be an important factor in certain healthcare jurisdictions.

## Introduction

The presence of a conduit between the middle ear and the airway was known as early as the 4th century BC. The term, Eustachian tube (ET), was named after a 16th century Italian anatomist- Batolomeo Eustachi, who was credited with its anatomical description. However, while Eustachi suggested that the ET serves solely as a drainage of middle ear (ME) secretions, over a century later, Duverney described the other ET function, which is to equalize ME air pressure. Valsalva suggested in 1704, that the ET is not permanently open, but is rather under muscular control (1). OETD is common and pivotal in many otologic pathologies, including middle ear atelectasis, acquired cholesteatoma formation and more (2). The commonly posited hypothesis is the causal effect of OETD in creating a negative middle ear pressure relative to ambient pressure, resulting in tympanic membrane atelectasis and retraction pocket formation. Failure to evacuate accumulated keratinized squamous cell epithelium from the lateral layer of the tympanic membrane is believed to be fundamental to cholesteatoma genesis (3). The first direct treatment of the ET in the form of an ET catheterization was attempted orally by Guyot, in 1724. (Guyot, 1724) and via the nasal cavities, by Cleland, and Wathen (in 1741 and 1756, respectively). In 1960, Toynbee realized that the ET is closed at rest, and opens briefly during swallowing. He also suggested constant absorption of ME air by the lining mucosa of the ME cleft, which was proven by Politzer in 1962. With the introduction of endoscopy and high resolution radiological imaging studies, we begin to refine our understanding of the dynamic functions of the ET, which continues to confound us. In simplistic terms, ET dysfunction (ETD) can be categorized as inappropriate closure, or abnormal patency. In the former, a subset of patients are identified as having obstructive ETD (OETD). Presenting symptoms characterized by aural fullness, hearing loss, otalgia, muffled hearing and tinnitus (4), with a prevalence of ∼1% of the adult population (5). Several independent risk factors were identified, including smoking (6), obstructive sleep apnea (7), sinusitis (8) and gastroesophageal reflux disease (9), and the overall ETD prevalence is 4.4%–4.6% (10, 11).

Traditionally, OETD is managed with little success, as medical therapies and eustachian tube auto-insufflation techniques are of limited value. Several treatment strategies exist for OETD: When risk factors addressed are not enough to control OETD symptoms, repeated Valsalva maneuvers with or without topical/systemic steroidal treatment and nasal irrigation can be used, with various success (12–15). When non-invasive treatment fails, tympanostomy tube can alleviate symptoms by equalizing the middle ear and ambient pressure, bypassing the ET, but not without downsides (16). Tympanostomy tube is reserved for more advanced disease and can be associated with short and long-term adverse effects, including foreign body reaction, water contamination leading to increased risk of otitis media, progressive thinning and perforation of the tympanic membrane, and more (17). In recent years, endoscopic dilatation devices have been developed to address OETD. This technique can be viewed as an extension of OETD management evolution, initiated by blind catheterization in the early 1700th (18), followed by catheterization with irrigation in the mid-1700th (19) and reinvigorated by the experience of sinus balloonplasty (20). The concept of BDET is to exert pressure injury to mucosal and deeper soft tissues in order to achieve a prolonged period of patency (21). Since the introduction of balloon dilatation Eustachian tuboplasty (BDET) in 2010 by Ockermann et al. (22), and the publication of a randomized controlled trial proving the superiority of BDET with pharmacotherapy over pharmacotherapy alone (23), it became standard of care for OETD patients who fail to recover completely after pharmacological treatment (15). There is a paucity of studies regarding the histological and physiological changes to the ET and the middle ear following balloon dilatation (21, 24, 25). But there has been sufficient literature in case series and multi-center trials in support of its benefit in selected individuals (26–28). Specifically, those with milder ET dysfunctions and barometric/altitude symptoms realize more benefit (29, 30). BDET in the context of existing middle ear disease (serous otitis, severe tympanic retraction, cholesteatoma) may be limited (15, 31).

The use of BDET as an adjunct to other otological procedures (tympanoplasty/mastoidectomy) for recurrent otitis media, and chronic suppurative otitis media also lacks evidence to support its use (32).

Traditionally, BDET is performed under general or local anesthesia, using a dedicated single-use non-compressible balloon, with an average procedural cost of ∼6,000 USD per candidate (33). To reduce material/device costs, an endovascular (EVB) BDET, which is “off-label” for this indication, was carefully studied for feasibility and safety, initially on cadavers (34), followed by humans (35), with promising results. Procedural cost can be further reduced by performing BDET using EVB under local anesthesia. The aim of this manuscript is to report our selection criteria, anesthesia protocol and outcome for BDET under local anesthesia using an off-label endovascular balloon and to compare our results to previous reports of local anesthesia BDET using the traditional approved balloons.

## Methods

### Methodology—case series

#### Ethical consideration

This prospective clinical pilot study was approved by the Sunnybrook Research Institute ethics committee (SUN-3156). Each patient provided informed consent to participate.

### Participants

Adult patients referred and diagnosed with OETD were offered to be enrolled in this study.

1. Diagnosed with unilateral or bilateral OETD for at least 3 consecutive months. Diagnosis was established by an average Eustachian tube dysfunction questionnaire-7 (ETDQ-7) score ≥3.
2. Refractory to pharmacotherapy that included either: 4 weeks of daily intranasal steroidal spray, or one completed course of oral steroids, within 3 months before study enrollment, with nasal irrigation.

Patients with a history or symptoms of the following conditions are excluded from the study: prior ET intervention, presence of tympanostomy tube, tympanic membrane (TM) perforation ipsilateral to the OETD, patulous ET, chronic otitis media, cholesteatoma, Meniere's disease, superior canal dehiscence, or temporomandibular joint disorder. Additionally, patients with uncontrolled rhinosinusitis, gastroesophageal reflux disease, active acute upper respiratory tract infection, cystic fibrosis, ciliary dysmotility syndrome or systemic immunodeficiencies were excluded. Extrinsic ET compression, cleft palate, prior radiation to the head and neck, craniofacial syndromes and 3 months history of head and neck surgery are also reasons for exclusion. Patients with ipsilateral non-favorable nasal anatomy, or carotid canal dehiscence based on CT scan were also excluded.

### Study design

After chronic OETD was diagnosed and informed consent was obtained, patients were screened for baseline performance in terms of otoscopy, nasal endoscopy, tympanometry, audiometry and ETDQ-7. ETDQ-7 was established by McCoul et al. on 2012 (36). Since then, it was validated to many languages (33, 37–42) and became the primary tool for ETD diagnosis and follow up. Available CT scans were reviewed for intranasal structure abnormalities and any carotid canal dehiscence. Patients were given 4 weeks of daily nasal saline irrigation unless used before enrollment. Then, BDET was performed, followed by 4 weeks of 3–4 daily nasal saline irrigation. Patients were followed up at 6 weeks and 6 months post BDET for all these measures. Primary outcome measures were defined as mean change in overall ETDQ-7 from baseline to 6 months and any BDET-related complication. Secondary outcome measures were changes in tympanometry, degree of tympanic membrane retraction/atelectasis and their response to the Valsalva maneuver, as well as a change in the pure tone average of four frequencies 500, 100, 2,000, and 4,000 Hz (PTA4). TM retraction was classified according to Sade classification (43), in which stage 1 is mildly retracted TM, stage 2 is retracted TM as medially as the incudo-stapedial joint, stage 3 is retracted TM over the promontory, and stage 4 is adhesive atelectatic TM. For objective tympanometry, improvement was defined per ear as a change from type B to type A or C, or from type C to type A. Subjective improvement was defined as an average ETDQ-7 score <3 points at the 6 months follow-up.

### Anesthesia protocol and surgical process

**Figure 1:**
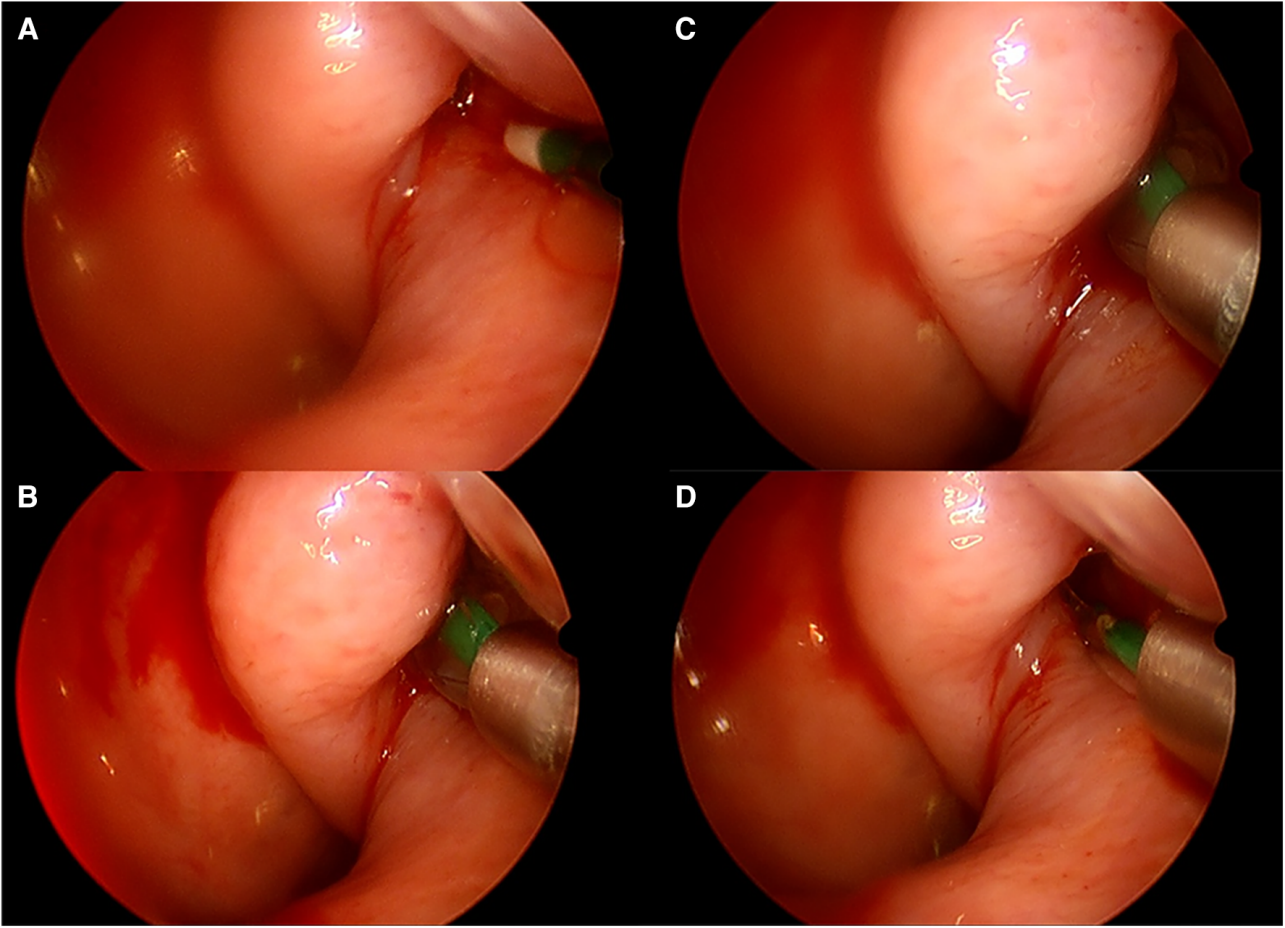
Intraoperative endoscopic (30°) view of left BDET. (**A)** Catheter tip is in front of ET orifice; (**B**) balloon in inserted, and partially inflated; (**C**) balloon is fully inflated; (**D**) balloon in deflated and removed.

The description of technical procedure of BDET has been described extensively in the literature, while the technique to use EVB has been previously published (34, 35). Patients were instructed to fast for 2 h before the procedure. Patients were positioned at 45° angle. Two sprays of oxymetazoline were given to each nostril, followed by cottonoids soaked with tetracaine 2% left for 15 min in each nasal cavity. No systemic steroids, anxiolytics or vestibular suppressants were given. The ETs or tympanic membranes were not anesthetized. Before the procedure, each endovascular balloon (EVB: Advance 35LP, COOK Medical, Bloomington, IN) was tested for leaks after all air was removed from its system. Under 30° angled nasal endoscope, a deflated endovascular balloon of 20 mm in length and 6 mm in diameter was carefully introduced through a maxillary sinus suction to the ET lumen, until the distal end (silver marker) of the deflated balloon was identified in the ET meatus. The operator verified full EVB insertion by direct endoscopic visualization of the proximal silver marker being held at the ET meatus (torus tobarius) (Figure 1D). With the silver marker at torus tobarius, the balloon length would start from there and go up by 2 cm inside cartilaginous portion of ET. The EVB was never advanced against pressure or resistance, and tended to slightly slide out upon inflation. Out-fracture of the inferior turbinate was performed if necessary. When the ventilation port was connected to suction to remove topical anesthetic solution or mucous from the nasal cavity, it was disconnected before inserting the endovascular balloon into the ET to allow ventilation and avoid positive middle ear pressure during balloon insertion into ET. The endovascular balloon was then inflated by saline to 12 ATM for 2 min. The inflation rate was constant at approximately 1 ATM/s to avoid rapid middle ear pressure change and resultant otalgia and possible vertigo. Upon ET dilation for 2 min, the endovascular balloon was gently deflated and removed from the ET. The inferior turbinate was repositioned if necessary. The endovascular balloon was examined for kinks *ex vivo*. Patients were instructed to avoid the consumption of hot food or liquids for a day, to reduce the risk of epistaxis from minor mucosal laceration, as well as to avoid sneezing against closed nostrils to avoid submucosal and subcutaneous emphysema. Intraoperative endoscopic photos are found in Figure 1.

### Statistical analysis

Categorical variables were summarized as frequencies and percentages. Continuous variables were evaluated for normal distribution by the use of histograms. The Chi-square and Fisher tests were used to compare categorical variables, and the Mann–Whitney test was used to compare continuous variables. All statistical tests were two-sided, and *P* < .05 was considered significant. SPSS software was used for all statistical analyses (IBM SPSS Statistics for Windows version 25, IBM Corporation, Armonk, NY, USA, 2017).

### Systematic literature review

This systematic literature review adheres to the Preferred Reporting Items for Systematic Reviews and Meta-Analyses (PRISMA) guidelines (44).

### Ethical consideration

This systematic literature review of collective published data did not require approval from the institutional review board or the ethical committee according to local law because it does not use individualized patient data.

### Search strategy

A comprehensive review of the scientific literature was conducted by means of an a-priori research protocol. We searched for articles in “Medline” via “PubMed”, “EMBASE”, and “Web of Science” without limitation of publication date up to March 1st, 2023. The exact search algorithm is shown in Supplementary I. Shortly, the Boolean operators used were either term [Eustachian tube], [Eustachian tuboplasty], [Balloon dilation tuboplasty], [Eustachius] or [Balloon tuboplasty], combined with the term [In office], [Local anesthesia] or [Awake], that appeared as a text or keyword anywhere in the paper. After all, publications had been identified, two investigators (OJU and MDB) independently excluded duplicate titles and then screened publications for suitability by consensus.

### Data extraction and quality assessment

The included articles described BDET under local anesthesia, with or without anxiolytics Benzodiazepine (midazolam) medication. In papers describing BDET under local and general anesthesia, only the local anesthesia arm was extracted for data synthesis. The target population was restricted to adults (≥18 years). Case series and clinical trials were enrolled if a minimal number of 5 subjects were included. Primary outcomes for systematic review were mean change in overall ETDQ-7 (or non-English validated form) and complication rate. The need to induce general anesthesia to complete the procedure or abortion was defined as a complication. Publication, cohort and data collection times were not restricted, nor were the clinical settings (outpatient clinic, hospital department). Studies that were not in English and those that involved nonhuman studies or were review articles, abstracts, or letters were excluded. Also excluded were studies that lacked descriptions of treatment protocols or treatment outcomes. (Supplementary II). The abstracts and articles of the identified papers were reviewed to determine which investigations met the above selection criteria for inclusion in this study. Two investigators independently extracted the original data (OJU and MDB).

After the enrollment process was completed, a manual review of all the references cited in the enrolled studies was performed in search of additional papers for inclusion. This process was performed to allow studies which had not been identified according to our search algorithm to be identified and assessed for suitability for inclusion and was proved to be an effective method for the identification of additional papers for systematic literature reviews in otology (45–48). A quantitative data sheet was constructed, and each relevant publication was analyzed regarding study design, demographic data, selection criteria, local anesthesia protocol, balloon used and outcomes. All publications were assessed independently by two coauthors for the risk of bias using ‘‘The Cochrane Collaboration's tool for assessing the risk of bias in randomized trials” (49) and “Newcastle–Ottawa Quality Assessment Scale criteria” (50), as shown in Supplementary III. Reconciliation of disagreements was performed by a third coauthor, who was blind to the other authors’ assessments.

## Results

### Systematic literature review

**Figure 2:**
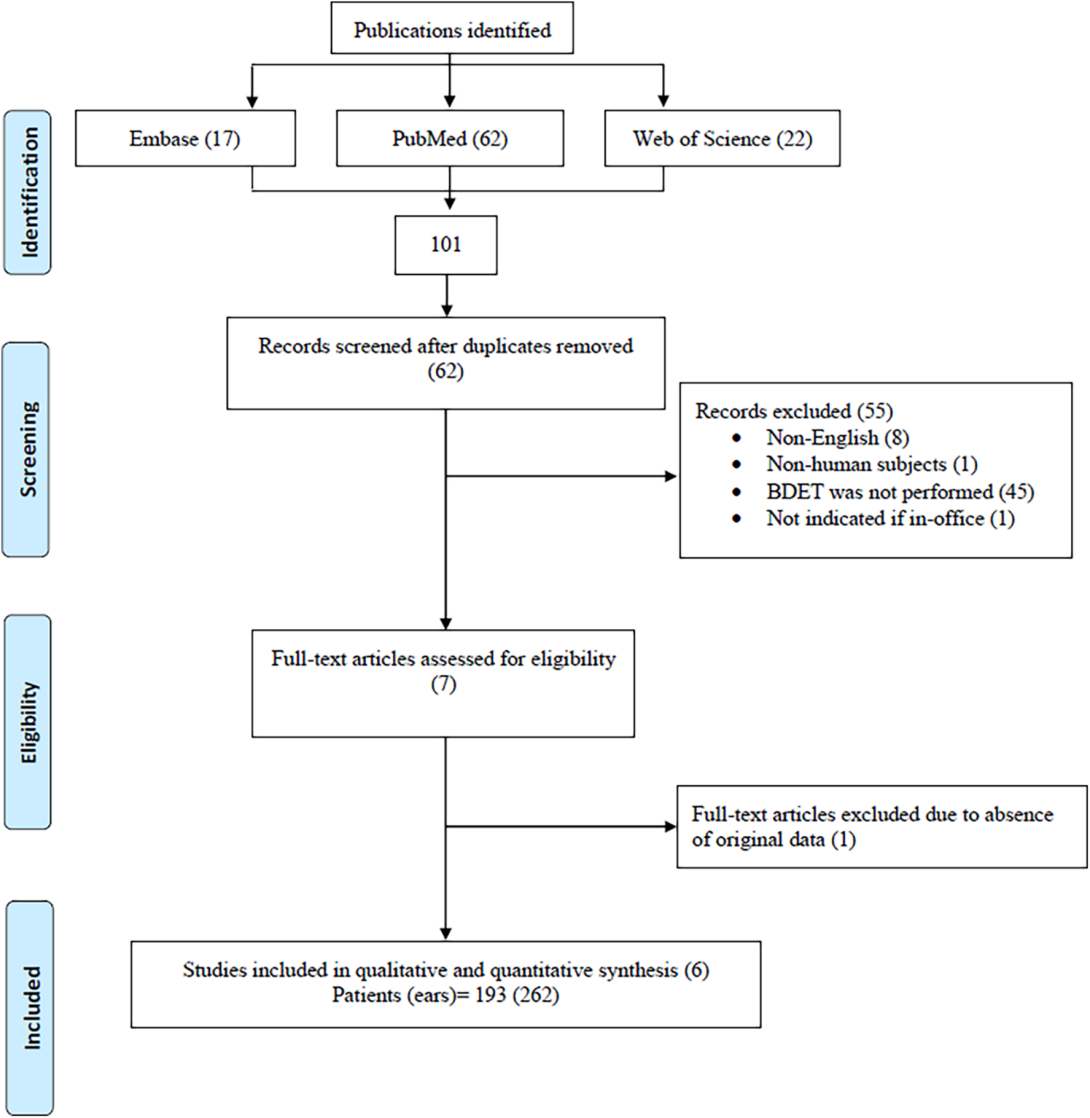
Flowchart of identification process of enrolled papers in the systematic literature review.

The literature review yielded 101 publications that met our search protocol. After 39 duplications were removed, 62 publications remained for screening. An additional 8 publications were excluded due to language other than English. Two more papers were excluded because of non-human subjects and the absence of indicating a BDET under local anesthesia (1 each). Additional 45 papers were excluded because BDET was performed under general anesthesia or because it was impossible to extract the clinical data of the local anesthesia BDET group from the total cohort. The resultant 7 publications were accessed for full text. One paper was excluded because of the absence of original data, leaving 6 papers for inclusion in the systematic literature review (Figure 2).

Five of the included papers were case series and one randomized controlled trial. Altogether, 193 patients (262 ETs) were included. Overall gender distribution could not be assessed due to missing reports in 3 papers (15, 31, 32), but 47/101 (47%) were males from the papers enrolled. Averaged (±SD) age ranged from 42 (±13) to 57 (±14). Indications for BDET widely varied: 2 papers did not report BDET indications (32, 51); out of the other 4, combinations of symptoms and tympanogram, ETDQ-7 and tympanogram, respectively, ETDQ-7 and symptoms and ETDQ-7 alone were used. ETDQ-7 mean item score for ETD diagnosis was not uniform, ranging from 2.1 to 3.0. Demographics and BDET criteria of published case series and our newly published case series are shown in Table 1 for the ease of comparison.

**Table 1:** Demographics and OETD criteria in the systematic literature review.

Author(s)	Years	Design	Number of patients (ears)	Males (%)	Age, average (±SD)	Bilateral (%)	Indications for BDET
Toivonen et al. (52)	2013–2018	Case control	58 (107)	24 (41)	57 (±14)	49 (85)	3 months of: [AF + HL + Tympanogram B/C] OR [AF + otalgia on barochallange + Tympanogram A]
Chen et al. (53)	2015–2019	Case control	25 (32)	13 (52)	42 (±13)	7 (28)	3 months of: ETDQ-7 score ≥2.1+ exclusion pf PET
Dean (54)	NA	Case series	33	NA	NA	NA	3 months of: [ETDQ-7 >2.2 + tympanogram B/C]
Luukkainen et al. (55)	NA	Case series	18 (27)	10 (56)	NA	9 (50)	NA
Meyer et al. (56)	2015–2016	RCT	38	NA	NA	NA	12 months of: [ETDQ-7 ≥3 + 3 or more of: otalgia, ear pressure, tinnitus, muffled hearing, clogged ears]
Luukkainen et al. (55)	NA	Case control	13	NA	48 (±4)	NA	NA
Ungar et al. 2024^a^	2021–2023	Case series	8 (12)	5 (63)	48 (±15)	4 (50)	3 months of: [ETDQ-7 >3] resistant to nasal irrigation and steroids

AF, aural feullness; HL, hearing loss; PET, patulous eustachian tube; RCT, randomized controlled trial; NA, not available.

^a^ This is the current newly published case series.

Eligibility for local anesthesia was missing in all but one paper (52). In this paper, only patients who tolerated the pre-procedural endoscopic exam well, had straightforward nasal anatomy, appropriate body habitus and absence of potentially exacerbated medical condition were enrolled. 6 dilatory balloon systems were used, making a comparison between them impossible. Anesthesia protocol varied as well. Five studies used systemic analgesia/anxiolysis and topical anesthesia. Topical anesthesia alone was used in one study (53). The nasal cavities were most commonly anesthetized by cottonoids soaked with a combination of tetracaine or lidocaine + decongestant like adrenaline or pseudoephedrine. The ET orifice was anesthetized in 5 papers with lidocaine cream ± tetracaine or pilocarpine. The tympanic membrane was anesthetized in 2 papers with 7% Tetracaine/Lidocaine. The anesthetic protocol and dilatory system used are shown in Table 2.

**Table 2:** Balloon dilation system and anesthesia protocol.

Author(s)	Balloon used	Systemic	Nostril	ET orifice	Otic	Outcome
Toivonen et al. (52)	6 mm sinuplasty balloon (Acclarent, Irvine, CA)	10 mg diazepam	Oxymethazoline 4% spray	7% tetracaine/lidocaine	7% tetracaine/lidocaine	Tympanogram, otoscopy, audiogram, need for revision at 6 month post-op
	6 mm Acclarent Aera balloon (Acclarent, Irvine, CA)		Cottonoid 2% tetracaine			
Chen et al. (53)	3.28 mm Bielfeld Dilation System (Spiggle & Theis, GmbH	–	Cottonoid 1% lidocaine + 0.01% adrenaline	Lidocaine crean	–	ETDQ-7 score, intraoperative pain and discomfort, willingness tochoose local anasthesia again
Dean (54)	Acclarent Aera balloon (Acclarent, Irvine, CA)	Diazepam	Oxymethazoline 4% spray	Cottonoids 7% tetracaine/lidocaine	7% tetracaine/lidocaine	Tympanogram
			Cottonoid 2% tetracaine			
Luukkainen et al. (55)	TubaVent	Fentanyl	Cottonoids 200 mg cocaine in 1 ml 0.1 adrenaline	25/25 mg lidocaine/pilocarpine cream (EMLA)	–	Pain and vital signs
	TubaVent short	Midazolam		20 mg cocaine in 1 ml 0.1 adrenaline		
		Propofol				
Meyer et al. (56)	XprESS ENT dilation system (Enthelus Medical, Plymouth, MN)	Diazepam	Lidocaine/neosynephrine spray	–	–	ETDQ-7 score, complication rate, tympanogram, otoscopy
			Pledgets 1:1,000 epinephrine/1% lidocaine			
			Injection 1:1,000 epinephrine/1% lidocaine			
Luukkainen et al. (57)	6 mm Acclarent Aera balloon (Acclarent, Irvine, CA)	1–2 g paracetemol	Xylomethazoline drops	25/25 mg lidocaine/pilocarpine cream (EMLA)	–	Pain and willingness to choose local anesthesia BDET again
		7.5 mg Midazolam				
		20–25 µg fentanil	Cottonoid 50 mg cocaine in 1 ml 0.01% adrenaline			
		Diazepam				

The most common outcome measured used was a tympanogram (3 papers), followed by ETDQ-7 (2 papers). Non-otologic outcome measures alone (pain, vitals and willingness to choose BDET under local anesthesia again) were present in 2 papers. Some papers compared the outcome measures between local vs. general anesthesia, while others compared baseline to follow-up performance. As a result, pooling outcome is impossible (Table 3).

**Table 3:** Outcome measures in systematic literature review.

Author(s)	Tympanogram	ETDQ-7	Atalectasis	Valsalva	Complication	Revision
Toivonen et al. (52)	+		+	+		+
Chen et al. (53)		+		+	+	
Dean (54)	+				+	
Luukkainen et al. (55)	Non- otologic outcomes					
Meyer et al. (56)	+	+	+	+	+	+
Luukkainen et al. (57)	Non- otologic outcomes					

The results of a bias risk assessment for the non-randomized trials (5 trials, at least 112 ears) and randomized controlled trials (1 trial, at least 38 ears) show low bias risk in all included papers. Supplementary III displays the stratification of each bias category.

## Current case series

Eight BDET candidates (12 ears) were enrolled (M: *F* = 5:3). Average (±SD) age was 48 (±15). Laterality is distributed equally. Independent risk factors for OETD included 2 active smokers, and one patient with controlled gastroesophageal reflux disease with proton pumps inhibitors and lifestyle modification. The most common presenting symptom was aural fullness (9/12), followed by ear pressure (7/12), hearing loss (5/8) and tinnitus (4/12). Otalgia was reported by 1 candidate. Pre-operatively, tympanic membrane retraction was evident in 10/12 patients, most of whom were Sade stage II (4/10), meaning that the TM retracted onto the incudo-stapedial joint. Sade tage I and III tympanic membrane retractions were documented in 1 and 3 patients. In 10/12 ears, the affected tympanic membrane did not move laterally during Valsalva maneuver. Pre-operative tympanogram was type B and C in 7 and 5 ears, respectively. Average (±SD) pre- BDET PTA4 was 37 dB (±14). Flexible nasal endoscopy was normal in 3/8 patients. Cobble stoning was the most common pathological finding (3/9 ETs), followed by hyperemia and secretions from ET in 3 and 1 ETs, respectively. Baseline mean (±SD) ETDQ-7 scores were 4.4 (±0.4). Pre- and post-operative assessment is shown in Table 4.

**Table 4:** Patients’ demographics, pre-operative assessment and outcome.

Pre-operative assessment																					6 months post-operative follow up											
Patient	Age	Gender	Side	HL	AF	Otalgia	Ear pressure	Tinnitus	TM retraction classification^a^	TM movement in Valsalva	Tympanogram	PTA4 Pre- BDET	Endoscopy	ETDQ-7 scores Pre-BDET							Average	TM movement in valsalva	Tympanogram	PTA4- Post BDET	ETDQ-7 scores post-BDET							Average
1	23	M	L	+	+				I	+	B	43	Normal	4	3	2	3	3	3	4	3.1	+	A	27	2	3	1	2	3	1	2	2.0
2	59	M	R		+		+	+	II	−	B	25	Cobble stoning	4	2	5	6	5	7	2	4.4	+	A	56	1	2	1	3	2	1	1	1.6
			L	+				+	II	−	C	61	Cobble stoning	4	1	5	5	5	5	3	4.0	+	A	45	3	2	2	3	1	3	2	2.3
3	41	M	R		+		+		II	−	C	30	Hyperemia	5	4	6	4	4	3	2	4.0	−	A	28	2	2	1	1	1	3	2	1.7
			L		+		+		III	−	B	42	Hyperemia	5	5	4	3	4	3	3	3.9	−	B	25	2	3	3	2	1	4	2	2.4
4	30	F	R						II	−	B	59	Hyperemia	5	5	7	3	6	5	6	5.3	+	A	42	3	4	3	2	3	2	3	2.9
			L		+	+	+	+	None	+	B	20	Cobble stoning	5	4	4	5	4	7	3	4.6	+	A	22	1	1	2	2	1	3	2	1.7
5	54	F	R	+	+		+		II	−	C	25	Secretions from ET	6	3	4	5	6	5	4	4.7	−	B	21	4	5	4	6	5	5	4	4.7
6	57	M	L	+	+		+		II	−	C	28	Normal	6	5	5	6	6	6	6	5.7	+	A	31	1	1	1	2	3	3	1	1.7
7	72	F	R						III	−	B	50	Cobble stoning	5	6	4	6	3	2	4	4.3	+	B	90	1	4	3	2	3	1	2	2.3
			L	+	+		+	+	III	−	C	29	Cobble stoning	5	2	5	5	4	4	6	4.4	+	B	24	2	1	1	3	3	3	1	2.0
8	50	M	R		+				None	−	B	31	Normal	5	4	6	3	3	2	5	4.0	+	A	33	3	3	1	2	1	2	2	2.0

HL, hearing loss; AF, aural fullness; TM, tympanic membrane; BDET, balloon dilation Eustachian tuboplasty; ABG, air bone gap.

^a^ Sade and Berco (43).

Pain was experienced to minor extent throughout the procedure. Two patients reported mild pain during out fracture of the inferior turbinate. This was self-limiting and subsided before the end of the procedure. Additionally, 1 patient reported otalgia during EVB inflation, probably as the result of abrupt pressure change. This was modified by reduction of EVB inflation speed. One patient experienced mild post procedural epistaxis from head of the middle turbinate. This epistaxis resolved after several minutes of conservative management. Besides this minor complication, no other complications were recorded.

Six months otoscopy excluded TM perforation among our cohort. TM retraction Sade stage 1 was recorded in 6 patients, 4 and 2 of whom suffered stage 2 and 3 pre-procedural TM retractions, respectively. Two patients with pre-procedural Sade stage 3 TM retraction, showed stage 2 retraction at the end of the follow-up period. Post-operative tympanometry was A in 8 ears, and B in 4 ears. No C shaped tympanometry was recorded post-operatively. Two tympanic membranes showed a change of tympanometry from C pre-operatively to B. TM movement in Valsalva was noted in 2 TMs pre-operatively and 9 TMs post-operatively. Post-operatively, ETDQ-7 scores declined to 2.3 (±0.5). This difference was significant (*p* = 0.0014). The average (±SD) post procedural PTA was 37 dB (±20), which is not significantly (*p* = 0.988) different from the pre-procedural PTA4. One patient (#5) failed to improve subjectively and objectively. This patient suffered obstructive sleep apnea, which may be responsible to treatment failure.

## Discussion

OETD is common and pivotal in many otologic pathologies, including middle ear atelectasis, acquired cholesteatoma formation and more (2). The commonly posited hypothesis is the causal effect of OETD in creating a negative middle ear pressure relative to ambient pressure, resulting in tympanic membrane atelectasis and retraction pocket formation. Failure to evacuate accumulated keratinized squamous cell epithelium from the lateral layer of the tympanic membrane is believed to be fundamental to cholesteatoma genesis (3). Traditionally, OETD is managed with little success, as medical therapies and eustachian tube auto-insufflation techniques are of limited value. Tympanostomy tube is reserved for more advanced disease and can be associated with short and long-term adverse effects, including foreign body reaction, water contamination leading to increased risk of otitis media, progressive thinning and perforation of the tympanic membrane, and more (17). In recent years, endoscopic dilatation devices have been developed to address OETD. This technique can be viewed as an extension of OETD management evolution, initiated by blind catheterization in the early 1700th (18), followed by catheterization with irrigation in the mid-1700th (19) and reinvigorated by the experience of sinus balloonplasty (20). The concept of ETBD is to exert pressure injury to mucosal and deeper soft tissues in order to achieve a prolonged period of patency (21). There is a paucity of studies regarding the histological and physiological changes to the ET and the middle ear following balloon dilatation (21, 24, 25). But there has been sufficient literature in case series and multi-center trials in support of its benefit in selected individuals (26–28). Specifically, those with milder ET dysfunctions and barometric/altitude symptoms realize more benefit. BDET in the context of existing middle ear disease (serous otitis, severe tympanic retraction, cholesteatoma) may be limited (15, 31). BDET is often performed under general anesthesia with a high-cost, dedicated ET balloon dilation system. We aimed to study the safety and therapeutic profile of a low-cost EVB used for OETD under local anesthesia and to compare our results to the literature. As far as the authors know, this is the first study that combines local anesthesia with EVB for BDET.

### Safety of in-office BDET with EVB

In our case series, no major complications were recorded. BDET is a safe procedure. In their systematic literature review, Randrup and Ovesen reported minor self-limited complications following BDET in 2% of 1,800 BDETs, the most common of which was mild epistaxis (58). They also identified one report of a patient who suffered post-procedural C6–7 radiculopathy, which was related to neck extension, and probably is not related to the procedure itself. The bony part of the ET and the internal carotid artery (IAC) share a common bony wall, making the posteromedial aspect of the ET bony-cartilaginous junction a landmark to the IAC first genu (59, 60). This common bony wall is so thin that it can be easily medialized during balloon inflation, compressing the carotid canal with associated anoxic brain damage (61). This situation is further complicated by the fact that carotid canal dehiscence (CCD) is not uncommon (>7%) and that near-dehiscence can be interpreted as CCD in high-resolution CT (62). There are several FDA approved dedicated DBET systems in the market. TubaVent and TubaVent short (Spiggle & Theis, Germany) is 20 mm long. XprESS devices (Srtyker Corporation, Michigan, US) have several dedicated balloons, 8 or 20 mm long. Acclarent Aera Balloon (Acclarent, Irvine, CA) manufactures 16 mm length balloon devices. CCD is of clinical significance in the petrous portion, more than 20 mm way from the ET meatus. This observation highlights the need to avoid over insertion of the device up the ET. Because the OETD resides in the cartilaginous part of the ETD (63), a selective cartilaginous BDET reduces the risk of carotid canal compression. This is the rationale behind the importance of direct visualization of the dilation device in the ET orifice and avoiding advancing the suction guide to the ET lumen, blocking the vision of the dilation device. A recent study, including 510 BDETs, with a CCD incidence of 6.3%, reported no CCD-associated BDET complications, thereby concluding that “fear of injury to the internal carotid artery during balloon dilatation might be disproportionate”. However, since BDET is performed as an elective procedure, for quality of life- anoxic brain damage can be devastating sequala. In this term, in-office BDET seems to be safer because the conscious candidate can report real-time focal motor and/or sensory dysfunction.

Recently, a case report of iatrogenic sudden sensorineural hearing loss occurred during BDET (64). Likely pathophysiological mechanism suggested an inner ear concussion, or inner ear membrane injury from barotrauma. Other report of extensive cervico-facial emphysema and/or pneumo-mediastinum (65–67) also emphasizes that balloon insertion must be performed under utmost care, while the depth and speed of inflation must be rigorously monitored to avoid a sudden pressure change and tearing of mucosa.

The present study is an extension of a prior cadaver study demonstrating a proof of concept using the EVB (35). In our series of 20 patients using the EVB [ref#36 and this study], the risk profile and outcomes are similar to a systematic literature review, in which 262 BDETs were performed under local anesthesia (52–57). We believe there is clinical equipoise between an EVB and other commercially available BDET products,

### Cost of in-office BDET with EVB

Several dedicated dilation devices for BDET are Class II FDA-approved. These single- use devices are priced at over USD$1,000. The economy of scale in the pricing of the EVB has driven the cost to approximately USD $ 170 per device. This combined with diminished operating room resource in performing the procedure under local anesthesia within the ambulatory care setting, there would be considerable savings regardless of the type of healthcare delivery system in the context of cost-effectiveness. This is important when considering repeating the procedure for recurrent symptoms.

### The outcome of in-office BDET with EVB

The effectiveness of the EVB appears comparable to other commercially available, dedicated balloons. In our study, one patient (#5) did not improve objectively (tympanometry and TM movement under Valsalva). Patient #3 (left ear) did not recover subjectively and objectively (ETDQ-7 score, TM movement under Valsalva and tympanometry). This is congruent with the reported 70%–90% success rate of BDET performed under local anesthesia (56, 68). Under general anesthesia, 63%–80% report improvement of tympanogram, with inferior success rate when chronic serous or adhesive otitis media coexist (30). While in-office procedures can potentially reduce the success rate due to limited intra-operative patient cooperation, proper patient selection can mitigate this type of selection bias. However, the EVB was proved in our study and a previous one (35) to be as effective as the dedicated ET dilation systems under general and local anesthesia.

### Patient satisfaction

Only 2 studies evaluated patients' satisfaction with BDET under local anesthesia. Chen et al. (53) reported 96% willingness to choose local anesthesia again, including patients who underwent bilateral BDET. This observation is probably in line with Luukkainen et al. (55), who reported a low visual analog scale (VAS) for pain and discomfort (∼1 ± 0.4 and ∼1.3 ± 0.7 on a 0–10 scale). Our subjects reported a 100% (8/8) willingness to repeat the procedure under local anesthesia if needed. Overall, patient satisfaction is closely related to the local anesthetic protocol. There was a significant difference in the reported local anesthesia methods, with non-uniform tympanic membrane and nasal cavity anesthesia. Tympanic membrane anesthesia controls baroreceptor activation in the middle ear, mainly in the tympanic membrane. During BDET, middle ear pressure can increase to as high as 206 deka Pascal (16 mm of Mercury) during insertion and inflation and as low as 253 deka Pascal (19 mm of Mercury) during deflation (69), while an abrupt 50 deka Pascal (4 mm of Mercury) change is associated with significant discomfort in 14% of the population (70). Another issue is the inter-aural middle ear pressure gradient. While this gradient exceeds 500 deka Pascal (38 mm of Mercury), a common scenario in bilateral ETD, where the operated ear reaches a peak pressure during balloon inflation and the contralateral middle ear pressure is still in the negatives, alterno-baric vertigo can result (71, 72). While various vestibular suppressants can control this adverse effect (55, 73), we found that a slow inflation rate of 1 ATM/s is enough to control pain from abrupt pressure change and alterno-baric vertigo. Hereby making ototopical anesthesia and systemic vestibular suppressants- unnecessary. Slowly balloon inflation has the advantage of keeping the vaso-vagal reflex, resulting from rapid tympanic cavity inflation, un-activated, as reported in the previous series (57).

## Conclusions

EVB is a safe, off-label system for BDET in this case series study, which gives a similar outcome, and costs less than the dedicated approved devices. It can be used under local anesthesia for a selected population with OETD. Larger cohort is needed to define the overall effectiveness and efficacy profiles of EVB-based BDET.

## Data Availability

The original contributions presented in the study are included in the article/**Supplementary Material**, further inquiries can be directed to the corresponding author.

## References

[1] Valsalva AM. De aure humana tractatus…Interposita est musculorum uvulae atque pharyngis nova descriptio. Pisarius (1704).

[2] Semaan MT Megerian CA. The pathophysiology of cholesteatoma. Otolaryngol Clin North Am. (2006) 39(6):1143–59. 10.1016/j.otc.2006.08.003 17097438

[3] Olszewska E Wagner M Bernal-Sprekelsen M Ebmeyer J Dazert S Hildmann H Etiopathogenesis of cholesteatoma. Eur Arch Otorhinolaryngol. (2004) 261:6–24. 10.1007/s00405-003-0623-x 12835944

[4] Schilder AGM Bhutta MF Butler CC Holy C Levine LH Kvaerner KJ Eustachian tube dysfunction: consensus statement on definition, types, clinical presentation and diagnosis. Clin Otolaryngol. (2015) 40(5):407. 10.1111/coa.12475 26347263 PMC4600223

[5] Ramakrishnan N D’Souza R Kadambi P. A systematic literature review of the safety and efficacy of eustachian balloon tuboplasty in patients with chronic eustachian tube dysfunction. Indian J Otolaryngol Head Neck Surg. (2019) 71:406–12. 10.1007/s12070-019-01637-7 31559212 PMC6737097

[6] Dubin MG Pollock HW Ebert CS Berg E Buenting JE Prazma JP. Eustachian tube dysfunction after tobacco smoke exposure. Otolaryngol Head Neck Surg. (2002) 126(1):14–9. 10.1067/mhn.2002.121320 11821759

[7] Magliulo G de Vincentiis, M Iannella G Ciofalo A Manno A Pasquariello B Eustachian tube evaluation in patients with obstructive sleep apnea syndrome. Acta Otolaryngol. (2018) 138(2):159–64. 10.1080/00016489.2017.1385846 28990834

[8] Tangbumrungtham N Patel VS Thamboo A Patel ZM Nayak JV Ma Y The prevalence of eustachian tube dysfunction symptoms in patients with chronic rhinosinusitis. Int Forum Allergy Rhinol. (2018, May) 8(5):620–3. 10.1002/alr.22056 29227048

[9] Brunworth JD Mahboubi H Garg R Johnson B Brandon B Djalilian HR. Nasopharyngeal acid reflux and eustachian tube dysfunction in adults. Ann Otol Rhinol Laryngol. (2014) 123(6):415–9. 10.1177/0003489414526689 24671547

[10] Kim AS Betz JF Goman AM Poe DS Reed NS Ward BK Prevalence and population estimates of obstructive eustachian tube dysfunction in US adolescents. JAMA Otolaryngol Head Neck Surg. (2020) 146(8):763–5. 10.1001/jamaoto.2020.0962 32496532 PMC7273314

[11] Shan A Ward BK Goman AM Betz JF Reed NS Poe DS Prevalence of eustachian tube dysfunction in adults in the United States. JAMA Otolaryngol Head Neck Surg. (2019) 145(10):974–5. 10.1001/jamaoto.2019.1917 31369057 PMC6681559

[12] Gluth MB McDonald DR Weaver AL Bauch CD Beatty CW Orvidas LJ. Management of eustachian tube dysfunction with nasal steroid spray: a prospective, randomized, placebo-controlled trial. Arch Otolaryngol Head Neck Surg. (2011) 137(5):449–55. 10.1001/archoto.2011.56 21576556

[13] Bellmunt AM Vila PM Chen JX Rosenfeld RM Hackell JM Shin JJ. Oral steroid usage for otitis media with effusion, eustachian tube dysfunction, and tympanic membrane retraction. Otolaryngol Head Neck Surg. (2016) 155(1):139–46. 10.1177/0194599816637845 27026728

[14] McCoul ED Megwalu UC Joe S Gray R O’Brien DC Ference EH Systemic steroids for otolaryngology–head and neck surgery disorders: an evidence-based primer for clinicians. Otolaryngol Head Neck Surg. (2022) 6:01945998221087664. 10.1177/01945998221087664 35349383

[15] Tucci DL McCoul ED Rosenfeld RM Tunkel DE Batra PS Chandrasekhar SS Clinical consensus statement: balloon dilation of the eustachian tube. Otolaryngol Head Neck Surg. (2019) 161(1):6–17. 10.1177/0194599819848423 31161864

[16] Adil E Poe D. What is the full range of medical and surgical treatments available for patients with eustachian tube dysfunction? Curr Opin Otolaryngol Head Neck Surg. (2014) 22(1):8–15. 10.1097/MOO.0000000000000020 24275798

[17] Mandel EM Casselbrant ML Kurs-Lasky M. Acute otorrhea: bacteriology of a common complication of tympanostomy tubes. Ann Otol Rhinol Laryngol. (1994) 103(9):713–8. 10.1177/000348949410300909 8085732

[18] Halsted TH. Pathology and surgery of the eustachian tube: with special reference to the value of closing it. Arch Otolaryngol. (1926) 4(3):189–95. 10.1001/archotol.1926.00590010207001

[19] Cleland A. Instruments to remedy some kinds of deafness. Philos Trans R Soc Lond. (1744) 41::848–51.

[20] Siow JK Kadah BA Werner JA. Balloon sinuplasty: a current hot topic in rhinology. Eur Arch Oto-Rhino-Laryngol. (2008) 265:509–11. 10.1007/s00405-008-0605-0 18299873

[21] Kivekäs I Chao WC Faquin W Hollowell M Silvola J Rasooly T Histopathology of balloon-dilation eustachian tuboplasty. Laryngoscope. (2015) 125(2):436–41. 10.1002/lary.24894 25154612

[22] Ockermann T Reineke U Upile T Ebmeyer J Sudhoff HH. Balloon dilatation eustachian tuboplasty: a clinical study. Laryngoscope. (2010) 120(7):1411–6. 10.1002/lary.20950 20564474

[23] Poe D Anand V Dean M Roberts WH Stolovitzky JP Hoffmann K Balloon dilation of the eustachian tube for dilatory dysfunction: a randomized controlled trial. Laryngoscope. (2018) 128(5):1200–6. 10.1002/lary.26827 28940574

[24] Christov F Gluth MB. Histopathology of the mucosa of eustachian tube orifice at the middle ear in chronic otitis media with effusion: possible insight into tuboplasty failure. Ann Otol Rhinol Laryngol. (2018) 127(11):817–22. 10.1177/0003489418796648 30187761

[25] Kim Y Kang JM Ryu DS Park JH Kang WS Park HJ. Serial histological changes in the cartilaginous eustachian tube in the rat following balloon dilation. PLoS One. (2022) 17(5):e0268763. 35613135 10.1371/journal.pone.0268763 PMC9132338

[26] Poe DS Silvola J Pyykkö I. Balloon dilation of the cartilaginous eustachian tube. Otolaryngol Head Neck Surg. (2011) 144(4):563–9. 10.1177/0194599811399866 21493236

[27] Silvola J Kivekäs I Poe DS. Balloon dilation of the cartilaginous portion of the eustachian tube. Otolaryngol Head Neck Surg. (2014) 151(1):125–30. 10.1177/0194599814529538 24705223

[28] McCoul ED Anand VK. Eustachian tube balloon dilation surgery. Int Forum Allergy Rhinol. (2012) 2(3):191–8. (Hoboken: Wiley Subscription Services, Inc., A Wiley Company). 10.1002/alr.21007 22253073

[29] Raymond MJ Shih MC Elvis PR Nguyen SA Brennan E Meyer TA A systematic review of eustachian tube procedures for baro-challenge eustachian tube dysfunction. Laryngoscope. (2022) 132(12):2473–83. 10.1002/lary.30132 35442523

[30] Sandoval M Navarro JJ Martínez-Beneyto P Herrera M Alfaro J López F Balloon eustachian tuboplasty for obstructive eustachian tube dysfunction: retrospective multicentre cohort study of 248 patients. Eur Arch Oto-Rhino-Laryngol. (2023) 2:1–11. 10.1007/s00405-023-07906-0 PMC10382357 36976369

[31] Si Y Chen Y Xu G Chen X He W Zhang Z. Cartilage tympanoplasty combined with eustachian tube balloon dilatation in the treatment of adhesive otitis media. Laryngoscope. (2019) 129(6):1462–7. 10.1002/lary.27603 30485447

[32] Ashry Y Kawai K Poe D. Utility of adjunctive procedures with balloon dilation of the eustachian tube. Laryngoscope Investig Otolaryngol. (2017) 2(6):337–43. 10.1002/lio2.110 29299505 PMC5743173

[33] Ungar OJ Cavel O Golan GS Oron Y Wasserzug O Handzel O. The hebrew version of the eustachian tube dysfunction questionnaire-7. Hearing Balance Commun. (2018) 16(2):114–9. 10.1080/21695717.2018.1463756

[34] Dahm V Chan HH Daly MJ Lui JT Lin VY Irish J The feasibility of eustachian tube dilation with a standard endovascular balloon: a comparative cadaver study. Otol Neurotol. (2022) 43(2):256–62. 10.1097/MAO.0000000000003404 34739430

[35] Dahm V Lui JT Jung S Lin VY Chen JM Le TN. The feasibility and safety of eustachian tube dilation with a standard endovascular balloon: a clinical pilot study. J Otolaryngol Head Neck Surg. (2023) 52(1):20. 10.1186/s40463-022-00599-1 36855202 PMC9976517

[36] McCoul ED Anand VK Christos PJ. Validating the clinical assessment of eustachian tube dysfunction: the eustachian tube dysfunction questionnaire (ETDQ-7). Laryngoscope. (2012) 122(5):1137–41. 10.1002/lary.23223 22374681 PMC3612400

[37] Alkholaiwi F Alnatheer AM Theyab RS Alyousef M Aldrees T Dahmash AB Cross-cultural adaptation, validation, and arabic translation of the eustachian tube dysfunction questionnaire (ETDQ-7). Int Arch Otorhinolaryngol. (2023) 26:636–42. 10.1055/s-0041-1740161 PMC9668431 36405457

[38] Özgür E Bilgen C Özyurt BC. Turkish validity and reliability of eustachian tube dysfunction questionnaire-7. Braz J Otorhinolaryngol. (2018) 84:435–40. 10.1016/j.bjorl.2017.05.001 28622915 PMC9449182

[39] Gallardo FP Onishi ET Lira FI Suzuki FB Testa JRG. Translation, validation and cultural adaptation of “the eustachian tube dysfunction questionnaire-7″ (ETDQ-7) to Brazilian Portuguese (BR). Braz J Otorhinolaryngol. (2019) 85(4):456–64. 10.1016/j.bjorl.2018.03.010 29753672 PMC9443040

[40] Hansen LJ Glad H Jørkov A Lundin K Kirchmann M. Validating the 7-item eustachian tube dysfunction questionnaire in Danish. Dan Med J. (2020) 67(7):A11190617. 32734886

[41] Lin WL Chou YF Sun CH Lin CC Hsu CJ Wu HP. Evaluation of thirty patients with eustachian tube dysfunction in Taiwan by questionnaire survey. J Formos Med Assoc. (2020) 119(2):621–6. 10.1016/j.jfma.2019.08.017 31540815

[42] Schröder S Lehmann M Sudhoff H Ebmeyer J. Assessment of chronic obstructive eustachian tube dysfunction: evaluation of the German version of the eustachian tube dysfunction questionnaire. HNO. (2014) 62:160–4. 10.1007/s00106-013-2764-6 24270966

[43] Sade J Berco E. Atelectasis and secretory otitis media. *Annals of Otology*. Ann Otol Rhinol Laryngol. (1976) 85(2_suppl):66–72. 10.1177/00034894760850S214 1267370

[44] Moher D Liberati A Tetzlaff J Altman DG; PRISMA group. Preferred reporting items for systematic reviews and meta-analyses: the PRISMA statement. PLoS Med. (2009) 6(7):e1000097. 10.1371/journal.pmed.1000097 19621072 PMC2707599

[45] Ungar OJ Shilo S Anat W Cavel O Handzel O Oron Y. Blast-induced cholesteatomas after spontaneous tympanic membrane healing. Ann Otol Rhinol Laryngol. (2019) 128(12):1147–51. 10.1177/0003489419865568 31366214

[46] Simani L Oron Y Handzel O Eta RA Warshavsky A Horowitz G Paper patching versus watchful waiting of traumatic tympanic membrane perforations: a meta-analysis. Laryngoscope. (2021) 131(9):2091–7. 10.1002/lary.29580 33881175

[47] Simani L Shilo S Oron Y Eta RA Handzel O Muhanna N Residual perforation risk assessment of intratympanic steroids via tympanostomy tube versus transtympanic injections. Laryngoscope. (2021) 131(9):E2583–91. 34002883 10.1002/lary.29609

[48] Ungar OJ Baris H Oron Y Shilo S Handzel O Warshavsky A Meta-analysis of time to extrusion of tympanostomy tubes by tympanic membrane quadrant. Clin Otolaryngol. (2021) 46(6):1165–71. 10.1111/coa.13843 34329540

[49] Higgins JP Altman DG Gøtzsche PC Jüni P Moher D Oxman AD The cochrane collaboration’s tool for assessing risk of bias in randomised trials. Br Med J. (2011) 343:d5928. 10.1136/bmj.d5928 22008217 PMC3196245

[50] Wells GA Shea B O’Connell D Peterson J Welch V Losos M *The Newcastle-Ottawa Scale (NOS) for assessing the quality if nonrandomized studies in meta-analyses*. Available online at: http://www.ohri.ca/programs/clinical_epidemiology/oxford.htm (accessed July 14, 2023)

[51] McCoul ED Weinreich HM Mulder H Man LX Schulz K Shin JJ. Utilization of invasive procedures for adult eustachian tube dysfunction. Otolaryngol Head Neck Surg. (2020) 163(5):963–70. 10.1177/0194599820931467 32525448

[52] Toivonen J Dean M Kawai K Poe D. Comparison of outcomes for balloon dilation of the eustachian tube under local vs. general anesthesia. Laryngoscope Investig Otolaryngol. (2022) 7(4):1120–8. 10.1002/lio2.842 36000054 PMC9392412

[53] Chen X Xie, L Zeng H Xu, Y Xiong H. Local versus general anesthesia for balloon dilation of the eustachian tube: a single-center retrospective study in a Chinese population. Ear Nose Throat J. (2020) 3:0145561320923172. 10.1177/0145561320923172 32425122

[54] Dean M. In-office balloon dilation of the eustachian tube under local anesthesia: a retrospective review. World J Otorhinolaryngol Head Neck Surg. (2019) 5(03):143–7. 10.1016/j.wjorl.2019.08.001 31750426 PMC6849361

[55] Luukkainen V Jero J Sinkkonen ST. Balloon eustachian tuboplasty under monitored anaesthesia care with different balloon dilation devices: a pilot feasibility study with 18 patients. Clin Otolaryngol. (2019) 44(1):87–90. 10.1111/coa.13236 30281926

[56] Meyer TA O’Malley EM Schlosser RJ Soler ZM Cai J Hoy MJ A randomized controlled trial of balloon dilation as a treatment for persistent eustachian tube dysfunction with 1-year follow-up. Otol Neurotol. (2018) 39(7):894. 10.1097/MAO.0000000000001853 29912819 PMC6075883

[57] Luukkainen V Kivekäs I Hammarén-Malmi S Rautiainen M Pöyhönen L Aarnisalo AA Balloon eustachian tuboplasty under local anesthesia: is it feasible? Laryngoscope. (2017) 127(5):1021–5. 10.1002/lary.26488 28409844

[58] Randrup TS Ovesen T. Balloon eustachian tuboplasty: a systematic review. Otolaryngol Head Neck Surg. (2015) 152(3):383–92. 10.1177/0194599814567105 25605694

[59] Ozturk K Snyderman CH Gardner PA Fernandez-Miranda JC. The anatomical relationship between the eustachian tube and petrous internal carotid artery. Laryngoscope. (2012) 122(12):2658–62. 10.1002/lary.23679 23161486

[60] Liu J Sun X Liu Q Wang D Wang H Ma N. Eustachian tube as a landmark to the internal carotid artery in endoscopic skull base surgery. Otolaryngol Head Neck Surg. (2016) 154(2):377–82. 10.1177/0194599815616799 26598497

[61] Abdel-Aziz T Schröder S Lehmann M Gehl HB Ebmeyer J Sudhoff H. Computed tomography before balloon eustachian tuboplasty–a true necessity? Otol Neurotol. (2014) 35(4):635–8. 10.1097/MAO.0000000000000214 24622017

[62] Moreano EH Paparella MM Zelterman D Goycoolea MV. Prevalence of carotid canal dehiscence in the human middle ear: a report of 1000 temporal bones. Laryngoscope. (1994) 104(5):612–8. 10.1002/lary.5541040515 8189992

[63] Poe DS Grimmer JF Metson R. Laser eustachian tuboplasty: two-year results. Laryngoscope. (2007) 117(2):231–7. 10.1097/01.mlg.0000246227.65877.1f 17277615

[64] Todt I Oppel F Sudhoff H. Sensorineural hearing loss after balloon eustachian tube dilatation. Front Surg. (2021) 8:615360. 10.3389/fsurg.2021.615360 33748180 PMC7973464

[65] Jang IJH Yuen HW. Extensive cervicofacial emphysema after eustachian tube balloon tuboplasty. Otol Neurotol. (2022) 43(9):e1056–7. 10.1097/MAO.0000000000003612 35877691

[66] Shah RR Thomas WW Naples JG Ruckenstein MJ. Subcutaneous emphysema and pneumomediastinum after eustachian tube balloon dilation. Otolaryngol Head Neck Surg. (2018) 159(1):203–5. 10.1177/0194599818768519 29759024

[67] Skevas T Dalchow CV Euteneuer S Sudhoff H Lehnerdt G. Cervicofacial and mediastinal emphysema after balloon eustachian tuboplasty (BET): a retrospective multicenter analysis. Eur Arch Oto-Rhino-Laryngol. (2018) 275:81–7. 10.1007/s00405-017-4805-3 29143098

[68] Catalano PJ Jonnalagadda S Vivian MY. Balloon catheter dilatation of eustachian tube: a preliminary study. Otol Neurotol. (2012) 33(9):1549–52. 10.1097/MAO.0b013e31826a50c3 23047259

[69] Sudhoff H Mittmann P Todt I. In vivo measurement of middle ear pressure changes during balloon eustachian tuboplasty. BioMed Res Int. (2018) 2018:45–9. 10.1155/2018/9519204 PMC6146622 30258853

[70] Schwanitz S Wittkowski M Rolny V Basner M. Pressure variations on a train–where is the threshold to railway passenger discomfort? Appl Ergon. (2013) 44(2):200–9. 10.1016/j.apergo.2012.07.003 22884634

[71] Kitajima N Sugita-Kitajima A Kitajima S. Altered eustachian tube function in SCUBA divers with alternobaric vertigo. Otol Neurotol. (2014) 35(5):850–6. 10.1097/MAO.0000000000000329 24751737

[72] Klingmann C Knauth M Praetorius M Plinkert PK. Alternobaric vertigo-really a hazard? Otol Neurotol. (2006) 27(8):120–1125. 10.1097/01.mao.0000235373.78116.a8 17130801

[73] Hain TC Yacovino D. Pharmacologic treatment of persons with dizziness. Neurol Clin. (2005) 23(3):831–53. 10.1016/j.ncl.2005.01.012 16026678

