# Supplementary material for: Balloon dilation of the eustachian tube using endovascular balloon under local anesthesia—a case series and systematic literature review
Source: Front Surg. 2024 Feb 20;11:1271248. doi: 10.3389/fsurg.2024.1271248 (PMC10912332; doi:10.3389/fsurg.2024.1271248)
Supplement: Supplementary file 1 [file Datasheet1.docx]

**Supplement I- Compete search algorithm used for this systematic literature review**

Search: **((Eustachian tube) OR (Eustachian tuboplasty) OR (Balloon dilatatory tuboplasty) OR (Eustachius) OR (Balloon tuboplasty)) AND ((In office) OR (Local anesthesia) OR (Awake))**

("eustachian tube"[MeSH Terms] OR ("eustachian"[All Fields] AND "tube"[All Fields]) OR "eustachian tube"[All Fields] OR ("eustachian"[All Fields] AND ("tuboplasties"[All Fields] OR "tuboplasty"[All Fields])) OR (("balloon"[All Fields] OR "balloon s"[All Fields] OR "balloons"[All Fields]) AND "dilatatory"[All Fields] AND ("tuboplasties"[All Fields] OR "tuboplasty"[All Fields])) OR "Eustachius"[All Fields] OR (("balloon"[All Fields] OR "balloon s"[All Fields] OR "balloons"[All Fields]) AND ("tuboplasties"[All Fields] OR "tuboplasty"[All Fields]))) AND ("office"[All Fields] OR "office s"[All Fields] OR "officer"[All Fields] OR "officer s"[All Fields] OR "officers"[All Fields] OR "offices"[All Fields] OR ("local anaesthesia"[All Fields] OR "anesthesia, local"[MeSH Terms] OR ("anesthesia"[All Fields] AND "local"[All Fields]) OR "local anesthesia"[All Fields] OR ("local"[All Fields] AND "anesthesia"[All Fields])) OR ("awake"[All Fields] OR "awakeness"[All Fields] OR "awakes"[All Fields] OR "awaking"[All Fields]))

**Translations**

**Eustachian tube:** "eustachian tube"[MeSH Terms] OR ("eustachian"[All Fields] AND "tube"[All Fields]) OR "eustachian tube"[All Fields]

**tuboplasty:** "tuboplasties"[All Fields] OR "tuboplasty"[All Fields]

**balloon:** "balloon"[All Fields] OR "balloon's"[All Fields] OR "balloons"[All Fields]

**tuboplasty:** "tuboplasties"[All Fields] OR "tuboplasty"[All Fields]

**balloon:** "balloon"[All Fields] OR "balloon's"[All Fields] OR "balloons"[All Fields]

**tuboplasty:** "tuboplasties"[All Fields] OR "tuboplasty"[All Fields]

**office:** "office"[All Fields] OR "office's"[All Fields] OR "officer"[All Fields] OR "officer's"[All Fields] OR "officers"[All Fields] OR "offices"[All Fields]

**local anesthesia:** "local anaesthesia"[All Fields] OR "anesthesia, local"[MeSH Terms] OR ("anesthesia"[All Fields] AND "local"[All Fields]) OR "local anesthesia"[All Fields] OR ("local"[All Fields] AND "anesthesia"[All Fields])

**awake:** "awake"[All Fields] OR "awakeness"[All Fields] OR "awakes"[All Fields] OR "awaking"[All Fields]
