# Supplementary material for: Balloon dilation of the eustachian tube using endovascular balloon under local anesthesia—a case series and systematic literature review
Source: Front Surg. 2024 Feb 20;11:1271248. doi: 10.3389/fsurg.2024.1271248 (PMC10912332; doi:10.3389/fsurg.2024.1271248)
Supplement: Supplementary file 2 [file Datasheet2.docx]

| Supplement II. Inclusion and Exclusion Criteria Used after Study Identification (PICOS). | | |
| --- | --- | --- |
| Variable | Included | Excluded |
| Target population | Adult humans populations | Pediatric population, non-humans |
| Intervention | BDET under local anesthesia | BDET under general anesthesia |
| Comparator | Success and complication rates | |
| Outcome | ETDQ-7 score, TM atelectasis, tympanometry, TM response to Valsalva, any complication including turning to general anesthesia and abortion. | |
| Time | Any | None |
| Setting | Any (outpatient clinic, hospital department) | None |
| Study design | Clinical trials with or without randomization, case series (if n≥5) | Meta-analyses, reviews, case reports |

BDET- Balloon dilation Eustachian tuboplasty; TM- Tympanic membrane; ETDQ-7- Eustachian tube dysfunction questionnaire 7.
