# Supplementary material for: Balloon dilation of the eustachian tube using endovascular balloon under local anesthesia—a case series and systematic literature review
Source: Front Surg. 2024 Feb 20;11:1271248. doi: 10.3389/fsurg.2024.1271248 (PMC10912332; doi:10.3389/fsurg.2024.1271248)
Supplement: Supplementary file 3 [file Datasheet3.docx]

Supplement III.

Risk of Bias Assessment (Newcastle–Ottawa Quality Assessment Scale Criteria).

| Author(s) (N of ears) | Representative of the exposed cohort | Selection of the non-exposed cohort | Ascertainment of exposure | Demonstration that outcome of interest was not present at start of study | Comparability of cohorts on the basis of the design or analysis | Assessment of outcome | Follow-up long enough for final  outcome assessment | Adequacy of follow-up of cohorts | Total score |
| --- | --- | --- | --- | --- | --- | --- | --- | --- | --- |
| Toivonen J et al, 2022 | 1 | 1 | 1 | 1 | 1 | 1 | 1 | 1 | 8 |
| Chen X et al, 2020 | 1 | 1 | 1 | 1 | 1 | 0 | 1 | 1 | 7 |
| Dean M, 2019 | 1 | 1 | 0 | 1 | 1 | 1 | 1 | 1 | 7 |
| Luukkainen V et al, 2019 | 1 | 1 | 0 | 1 | 1 | 1 | 1 | 1 | 7 |
| Luukkainen V et al, 2017 | 1 | 1 | 1 | 1 | 1 | 1 | 1 | 1 | 8 |

0- low bias risk for the respective category; 1- high bias risk for the respective category
*Very high risk of bias (0 to 3 points), high risk of bias (4 to 6 points), and low risk of bias (7 to 9 points).

The Cochrane Collaboration’s tool

| Author(s) | Random Sequence Generation  (Selection Bias) | Allocation Concealment (Selection Bias) | Blinding of Participants and Researchers (Performance Bias) | Blinding of Outcome Assessment  (Attrition Bias) | Incomplete Outcome Data | Selective Reporting (Reporting Bias) |
| --- | --- | --- | --- | --- | --- | --- |
| Meyer TA et al, 2018 | + | + | + | + | + | + |

(+) Low bias risk; (-) High bias risk; (?) Unclear bias risk
